# Impact of inclusive leadership on employees’ innovative behavior: A relational silence approach

**DOI:** 10.3389/fpsyg.2023.1144791

**Published:** 2023-03-06

**Authors:** Guo-feng Wu, Mei Li

**Affiliations:** University of Electronic Science and Technology of China Zhongshan Institute, Zhongshan, China

**Keywords:** inclusive leadership, relational silence, employee innovative behavior, China, Guangdong Province

## Abstract

**Introduction:**

Although employees’ silence is a common phenomenon in organizations, the mediating role of relational silence has not been studied in inclusive leadership and innovative behavior. In this study, based on the theory of social exchange, relational silence is used as a mediating variable to explore the internal mechanisms of inclusive leadership on employees’ innovative behavior.

**Methods:**

Data from 263 in-service leaders and employees were collected using convenience sampling and analyzed using Amos and SPSS statistical software package *via* questionnaires distributed to companies in six cities in the Guangdong province of China.

**Results:**

The results showed that inclusive leadership has a significant positive predictive effect on employees’ innovative behavior (*β* = 0.590, *p* < 0.01), while inclusive leadership is negative and significantly correlated with relational silence (*β* = −0.469, *p* < 0.01). More so, relational silence has a significant negative correlation with employees’ innovative behavior (*β* = −0.408, *p* < 0.01), and relational silence partially mediates the relationship between inclusive leadership and employee innovation behavior.

**Discussion:**

The mediating role of relational silence between inclusive leadership and employees’ innovative behavior is revealed for the first time, theoretically broadening and enriching the connotation of inclusive leadership’s influence mechanism on employees’ innovative behavior and providing new ideas in practice for constructing inclusive leadership styles, reducing the incidence of relational silence, and evoking employees’ innovative behavior.

## Introduction

Innovation is the engine that propels economic growth, and as the economic and technological landscape constantly changes, business competition is getting more ferocious. Businesses must continuously innovate to survive, develop and maintain core competitiveness (Scott and Bruce, 1994). Corporate innovation is based on individual employee innovation, which is essential for the company’s survival and growth. A feeling of innovation emerges only when employees believe that specific variables lead to inventive behavior. Scholars have now investigated the factors influencing employees’ innovative behavior concerning individual characteristics such as leadership style, relational conflict, and employee Silence, as well as organizational climate, social background, performance rewards, resource support, and organizational environment (Smith and Tushman, 2005; Siyal et al., 2021; Lee et al., 2022). Leadership style is considered one of the essential external motivating factors in the innovation process of employees (Siyal et al., 2021). Silent behavior is widespread in real-life circumstances where employees are unwilling to speak up about workplace difficulties. Employees, in most situations, choose to remain silent when presented with flaws or possible difficulties in production and operation for various reasons (Morrison and Milliken, 2000). Although research has indicated that employee silence has a detrimental influence on employee creativity, the literature has not yet reported on the mechanism of relational silence as a mediating variable to explore the effect of inclusive leadership on innovative employee behavior. The purpose of this study is to explore whether and how relational silence can play a mediating role between inclusive leadership and employee.

Some mediating variables, such as psychological empowerment (Javed et al., 2019; Cetinkaya and Yesilada, 2022), workplace friendship Berman et al., 2002, psychological safety (Brown and Treviño, 2006; Kark and Carmeli, 2009; Javed et al., 2019; Wang et al., 2021), intrinsic motivation (Siyal et al., 2021), Innovative self-efficacy (Tierney and Farmer, 2002; Javed et al., 2021; Wang et al., 2021), Team psychological safety (Edmondson, 1999), knowledge sharing (Lee et al., 2010), psychological capital (Fang et al., 2019), perceived organizational support (Qi et al., 2019), have been investigated in relation to the effects of inclusive leadership on employees’ innovative behavior. In order to take effective measures to improve employee innovation from various perspectives, it is vital to investigate new mediating variables that can have a mediating effect.

Employee silence has a negative impact in most situations (Vakola and Bouradas, 2005), leading to burnout, decreased satisfaction, decreased engagement, decreased motivation, and cognitive dissonance for the individual employee, as well as for the company, depriving managers of objective and realistic information about the problem, reducing the efficiency and quality of decision making and missing the opportunity to improve innovation. For businesses, this can deprive managers of objective and real-world knowledge about challenges, limiting decision efficiency and quality while also losing opportunities for progress and innovation (Morrison and Milliken, 2000). Employee silent behavior is influenced by various factors, which can be classified into three categories: personal, leadership, and organizational. Leadership factors (including leadership style, behavior, and member relationships) are among the most critical factors influencing staff silent behavior. Ethical leadership can lead to a harmonious interpersonal relationship between leaders and staff, affecting staff’s silent behavior (Brown et al., 2005; Brown and Treviño, 2006; El-Gazar and Zoromba, 2021). Going further, there is a fundamental notion of sincere leadership that juxtaposes the object of employees’ trust and dependence (Avolio and Gardner, 2005). Consequently, this makes employees express their honest views at the workplace due to an enticing influence of genuine leadership, which suppresses the generation of silent behavior in an organization (Monzani et al., 2016). However, the toxic leadership (Farghaly Abdelaliem and Abou Zeid, 2023), abusive leadership (Xu et al., 2015), and authoritarian leadership (Duan et al., 2018) styles make employees lean toward silence, to protect their interests. This contrasts with the inclusive leadership style, which prioritizes leadership behaviors such as openness, effectiveness, and accessibility (Qi et al., 2019), encompassing full interaction with subordinates, paying more attention to the needs and ideas of subordinates at the workplace, as well as, actively listening to the views of subordinates. Taken together, based on the theory of basic needs satisfaction, inclusive leadership can meet employees’ relationship needs and assist in promoting employees’ self-reporting behaviors, thus effectively reducing the frequency and extent of employees’ silent behaviors in an enterprise (Jolly and Lee, 2021).

The research on inclusive leadership and employee silence is in the exploratory stage. According to Brinsfield (2013), there are six dimensions of employee silence, which are as follows: defensive silence, relational silence, lack of confidence silence, biased silence, ineffective silence, and disengaged silence. This study explored the impact of inclusive leadership on Employees’ innovative behavior using a relational silence approach. Relational silence reflects the behavior that members of an organization exhibit when they sometimes choose to remain silent to maintain good interpersonal relationships at the workplace. Considering that Chinese traditional ideology and culture create a strong relationship culture among people, in several Chinese enterprises, the relationship between leaders and members is an essential component of organizational culture. To a large extent, the superior-subordinate relationship affects the behavior of employees, as exhibited by employee silence behavior. Moreover, no research has been reported on the mechanism of action between inclusive leadership and employee innovation behavior using relational silence as a mediating variable. In a context where Chinese companies are influenced by Confucianism, exploring the mediating effect of relational silence between inclusive leadership and employees’ innovative behavior can broaden and enrich the connotation of the mechanism of action of inclusive leadership on employees’ innovative behavior and provide new ideas for building inclusive leadership styles, reducing the occurrence of relational silence and maximizing the stimulation of employees’ innovative behavior.

## Theory and hypotheses

### Inclusive leadership and employees’ innovative behavior

The pros and cons of inclusive leadership style impacts on employees’ innovative behavior. Based on the contemporary leadership literature review, the typical behavioral characteristics of inclusive leadership are listening to employees’ views, tolerating employees’ views and failures, tolerating employees’ mistakes rationally, and encouraging and guiding employees when they make mistakes (Qi et al., 2019; Ma and Tang, 2022), willingness to listen carefully to the ideas of subordinates when they are doing a good job, as well as praising employees that perform excellently (Nembhard and Edmondson, 2006; Shafaei and Nejati, 2023). That said, inclusive leadership style can be described as a rich connotation of managerial leadership, in which subordinates have more interaction with the leadership team, coupled with good interpersonal communication with subordinates by paying attention to the needs of subordinates, as well as listening to their views *via* demonstrated openness, effectiveness, and accessibility (Hirak et al., 2012; Khan et al., 2022). In addition, this employee innovation behavior inculcates the idea that employees can produce new ideas in the workplace through active participation, the use of organizational resources to support these ideas, as well as *via* the implementation of plans to achieve innovative behaviors in employees (Scott and Bruce, 1994).

According to Shalley and Gilson (2004), leadership style is one of the most important factors influencing employees’ innovative behavior. Quite remarkably, encouraging inclusive leadership behavior holds the promise of improving the work experience of all staff members of an organization, thus, increasing their productivity, as well as stimulating innovative behavior at the workplace (Randel et al., 2018). Moreover, in recent years, inclusive leadership has become a new style of leadership, which can significantly predict employees’ innovative behavior. Similarly, the institution of an open environment at the workplace and the equal treatment of employees provided by generally inclusive leaders ensure that employees feel that they are being justly treated and valued, which in turn prompts employees to think creatively about the issues they face in organizational practice (Hollander, 1992). Besides, an inclusive leadership style characterized by openness, accessibility, and effectiveness can motivate employees to feel a sense of human-to-work fit, which in turn promotes employees’ well-being and innovative behavior (Choi et al., 2016; Fang et al., 2019). In real-life management practice, when the leader uses an inclusive management model the enterprise incentive mechanism is sound. In like manner, the internal personnel of such an enterprise reassures one another to appreciate the situation, by appreciating that it is advantageous for their organization to enhance the employee’s innovative behavior (Hollander, 1992). On top of that, an inclusive leadership style promotes cooperative behavior in the workplace, as well as grants employees the rights and freedom to manage complex situations through the enablement of employees to express their diverse opinions, thus tapping from and stimulating employees’ creative desires, reducing homogeneity, and also improving the innovation performance of enterprises (Choi et al., 2016; Qi et al., 2019). Ultimately, inclusive leaders not only appreciate their employees, they appreciate their work performance, and are willing to listen to their new ideas. Consequently, employees would work under inclusive leaders are encouraged to put forward their ideas and suggestions bravely, which in turn promotes and facilitates innovation in their firms (Javed et al., 2018; Elsaied, 2020). Recent related research further confirms that inclusive leadership significantly and positively predicts employees’ innovative behavior (Wang et al., 2021; Cetinkaya and Yesilada, 2022; Zhong et al., 2022). Based on the above analysis, it was considered necessary to propose the following hypothesis:

*Hypothesis 1*: Inclusive leadership has a positive impact on employees’ innovative behavior.

### Inclusive leadership and relational silence

As specified by Brinsfield (2013), relational silence refers to the behavior that employees choose to keep silent to protect the harmonious interpersonal relationship established during the communication process in their organization. As earlier stated, this concept also synthesizes the influence of antecedent motivation on employee silence, which leads to a further subdivision into six dimensions of employee silence. Although contemporary research focuses on employee silence, to the best of the researcher’s knowledge, there are very few studies on inclusive leadership and relational silence. This paper, therefore, employs the relational silence aspect of the employee silence approach, which refers to the behavior that employees choose to be silent to protect the harmonious interpersonal relationship at the workplace.

Nembhard and Edmondson (2006) argues that when leaders are open-minded, they see communication with their employees as being egalitarian, and also accept the differences that employees exhibit. Concurrently, when employees are aware of the amiable characteristics of leadership, the courage to offer a different opinion breaks the employee’s silence in an organization. Since inclusive leadership is open and accessible, allowing employees to realize that managers are ready to communicate with them and willing to listen to their opinions, employees’ silence can assume a relational dimension (Jolly and Lee, 2021). In this environment, managers communicate openly with their employees, promoting employee development, as well as enabling employees to express their views with confidence, which is conducive to improving employees’ psychological security, thereby reducing employee silence (Brown et al., 2005; Brown and Treviño, 2006; Alingh et al., 2019). Considering (Lee and Dahinten, 2021) research which studied the behaviors of nurses in hospitals, it was uncovered that the psychological safety of nurses was enhanced under the inclusive leadership style, and also, to improve the safety status of patients, the psychological safety of nurses is paramount and enhanced under an inclusive leadership style, thus, nurses were able to speak out and expose mistakes under inclusive leaders, Their research showed that inclusive leadership was negatively correlated with the intention to disclose errors and favorably correlated with speaking out. Therefore, when leaders treat employees in a more approachable manner, and contemporaneously have a higher degree of intimacy with employees, the quality of leader-member exchange relationships is high, and employees’ silent behavior is reduced (Detert and Burris, 2007; Xu et al., 2015; Jolly and Lee, 2021). Additionally, by taking the time to listen when staff members approach them with difficulties, leaders convey to staff that they are important and deserving of the leader’s time and attention, fulfilling the desire for relatedness (Ye et al., 2019). This, therefore, emphasizes that inclusive leadership embodies openness, accessibility, and listening to the views of employees, as well as the adoption of such behavior, which to a large extent can inhibit employees’ silent behavior. Based on the above analysis, the following hypothetical proposition is being investigated in this research:

*Hypothesis 2*: Inclusive leadership has a negative impact on relational silence.

### Relational silence and employees’ innovative behavior

It has been observed that employees’ forward-looking behavior is being pressured and influenced by individual aspirations, colleagues, and leaders, to avoid interpersonal conflicts at the workplace. Therefore, employees can choose to maintain the status quo and/or remain silent during critical situations in firms where they work. According to Lee et al. (2022) and Maqbool et al. (2019) employee silence also negatively affects employee innovation behavior. Employees’ Defensive Silence has a negative relationship with creativity (Jahanzeb et al., 2021). Employees’ silence-based behavior may deprive them of any chance to offer original contributions, which limits their creativity in work environments (Wynen et al., 2020). Generally, employees’ silence hinders the aggregate levels of development and innovation in an enterprise. If employees conceal their thoughts about organizational problems, the enterprise retrogressively misses out on most development opportunities that the business ecosystem offers. Concurrently, many studies have shown that employee silence can have several negative effects. For instance, employee silence behavior not only affects the organization’s planning and innovation performance but also causes employees to feel fatigued while at work (Morrison and Milliken, 2000). Also, employees’ silence can affect personal health, hygiene, and thinking, which triggers anxiety levels amongst employees, thus preventing them from changing or innovating in the firm (Avery and Quiñones, 2002). Concomitantly, silent behavior can as well affect employees’ emotional regulation, and the generation of vital psychological and physical changes, which leads to an increase in depression, and memory impairment, consequently lowering employees’ immunity levels, which also significantly impedes their perception of innovation and diminishes employees’ motivation to innovate (Morrison and Milliken, 2000).

Based on the theory of social exchange, most employees tend to exchange information when they all agree to abide by the principle of mutual benefit. Hence, the process of cost–benefit analysis ensures that anti-innovation behaviors such as silence can be reduced when there is an opportunity to exchange views with other employees. Comparatively, when employees do not have access to relatively scarce enterprise information, they find themselves in a disadvantageous position during organization-wide social relations. However, when employees have access to fresh ideas or put forward different voices in response to the current systems or concepts, the organization would benefit from a potential firm-wide increase in the level of opportunity for innovation. On the contrary, employee silence makes the organization receive less negative information. Thus, such an enterprise misses an opportunity to correct past mistakes, which, in turn, negatively affects the organization’s path toward progress (Nemeth, 1997). Based on the above analysis, the following hypothesis was put forward:

*Hypothesis 3*: Relational silence has a negative impact on employees’ innovative behavior.

### The mediating role of relational silence

Employees choose to remain silent at work for fear of damaging their relationships. Nonetheless, silence reduces positive communication among employees, and also hinders the exchange of information and ideas, as well as reduces the quantity and quality of knowledge transfer within an organization. Whiteside and Barclay (2013) find that tacit silence, inaction silence, and prosocial silence were significantly negatively correlated with employee well-being, but positively correlated with employee stress and turnover intention. In light of this phenomenon, employee silence is not conducive when cross-fertilizing ideas or adopting new knowledge within an organization. Just as it is not conducive when advancing organizational learning in firms. This is because it eventually hinders the occurrence of creativity within such organizations (Morrison and Milliken, 2000).

Furthermore, employee silence mediates the relationship between abusive management and employees’ creative performance (Lee et al., 2022). Nurses’ silence can mediate the link between toxic leadership and organizational performance (Farghaly Abdelaliem and Abou Zeid, 2023). Employees’ sense of supervisor rejection and their creativity are mediated by defensive silence (Jahanzeb et al., 2021). The findings of Broeng’s research suggest that tacit silence mediates some of the mechanisms by which organizational justice influences emotional exhaustion and withdrawal (Broeng, 2018). Besides, in the organizational justice field of study, it influences the physiological withdrawal behavior, as well as plays an intermediary role in the actual performance levels within an organization. Likewise, inaction silence mediates the effects of organizational justice on emotional exhaustion, psychological withdrawal, and physiological withdrawal. Moreover, if the key personnel and senior managers in an enterprise often ignore and reject suggestions made by their employees, it would increase the degree of the silence of the employees. However, the degree of employee silence is reduced when managers in an enterprise behave with openness, fairness, and trust, and shows enormous goodwill and appreciation for efforts made by employees that perform allocated tasks successfully (Vakola and Bouradas, 2005). Therefore, a high-quality inclusive leadership atmosphere within an organization can effectively restrain employees’ silent behavior, promote employees’ speech behavior, and improve the frequency and quality of leader-member exchange, thus in the process, synchronously stimulating employees’ innovative behavior. Based on the above analysis, the following hypothetical proposition is being investigated in this research:

*Hypothesis 4*: Relational silence mediates the relationship between inclusive leadership and employees’ innovative behavior.

## Research methodology

### Research model

**Figure 1:**
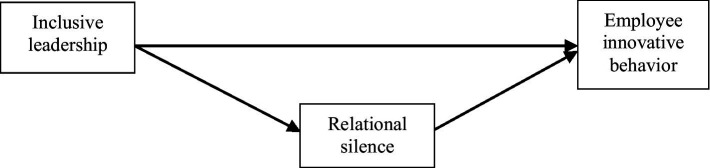
Research model.

Based on the above analysis, it is envisaged that inclusive leadership can positively influence employees’ innovative behavior, while relational silence plays a mediating role between inclusive leadership and employees’ innovative behavior. Accordingly, it is considered imperative to construct the following theoretical research model, to diagrammatically depict the abovementioned relationships (Figure 1).

### Sample

The convenience sampling method was used to collect data and test hypotheses; a combination of online and offline survey methods was used; The sample size was determined based on the research literature in psychology, the majority of which presented valid questionnaires in the region of 300. Most researchers have empirically calculated the number of questionnaires to be distributed as 10 times the number of items in a scale-based questionnaire. Because the scale in this study has 20 questions, at least 200 valid questionnaires were necessary. We predicted that the returned questionnaires’ return rate and validity rate would be around 80%, 400×80%×80% = 256; based on this calculation and analysis, it was determined to distribute 400 questionnaires. With a sample structure of the example structure was based on the working leaders and employees of four firms in each of Guangdong Province’s six cities. A total of 324 questionnaires were collected, indicating a recovery rate of 81 percent. After eliminating the incomplete and invalid questionnaires, a total of 263 effective questionnaires (49 in Guangzhou, 46 in Shenzhen, 40 in Dongguan, 42 in Foshan, 42 in Zhuhai, and 44 in Zhongshan) were obtained, indicating an effective rate of 81.17 percent.

Furthermore, the distribution of sample characteristics was as follows: 43.3 percent of the respondents were male, while the remaining 56.7 percent of the respondents were female; as per the age classification of respondents, 58.9 percent were 25 years or younger, 31.6 percent were aged between 26 and 35 years old, 6.8 percent were aged between 36 and 45 years old, and 2.7 percent of the respondents were either 46 years old or older. In terms of educational qualification, 4.2 percent of the respondents had either a high school education or less, 12.2 percent had a college education, 65 percent had a bachelor’s degree, and 18.6 percent had either a master’s degree or higher educational qualification. Likewise, the descriptive characteristics of the respondents’ working experience revealed that 56.3 percent of the respondents had less than one (1) year of working experience, 15.6 percent of the respondents had between 1 and 3 years of working experience, 9.9 percent of the respondents had 3.5 years working experience, 11.8 percent of the respondents had between 5 and 10 years working experience, while 6.5 percent of the respondents had above 10 years working experience. In terms of the rank of these officials, 63.5 percent of the respondents were ordinary staff, 20.2 percent of the respondents were junior managers, 14.1 percent of the respondents were middle managers, and 2.3 percent of the respondents were senior managers. Besides, in terms of job types, 6.1 percent of the respondents were engaged in production and workshop work, 34.6 percent of the respondents were engaged in finance, personnel, and administrative consulting work, 25.5 percent of the respondents were engaged in marketing and service, 17.1 percent of the respondents were engaged in technical operations, R & D and operations jobs, while 16.7 percent of the respondents worked in other positions.

### Variable measurement

To ensure the reliability and validity of the measurement instrument, the questionnaires used in this study employed the classic maturity scale. However. the study variables were measured on a continuum influence using a five-point Likert scale (where, 1 = completely disaccord, 2 = basically disaccord, 3 = general, 4 = basically accord, and 5 = perfect match.)

*Inclusive leadership:* Over and above that, inclusive leadership style was measured by a three-dimensional (openness, accessibility, and availability) nine-item scale proposed by Carmeli et al. (2010), as follows: “Leaders are willing to listen to my new ideas,” “Leaders focus on the opportunity to improve work.”

*Employee innovation behavior:* Besides, the measurement of employee innovation behavior is based on a well-developed and widely used scale developed by Scott and Bruce (1994), which consists of six items, such as “I like to explore in the work and learn new technology, new ideas,” “I will work hard to achieve new ideas, to develop the right opportunity for it.”

*Relational silence:* In addition, relational silence was measured using a five-item scale developed by Brinsfield (2013), which comprises questions such as “I want to remain silent to avoid hurting someone’s feelings.”

### Validity and reliability tests

To safeguard the validity and reliability of this study, several tests were carried out. Firstly, Cronbach’s alpha value of all the questionnaire items was 0.791 (which is above the recommended cut-off point of 0.7), and that of inclusive leadership was 0.875. For relational silence, Cronbach’s alpha value was 0.73, while Cronbach’s alpha value for employee innovation behavior was 0.834. Thus, all the questionnaire items Cronbach’s alpha values were all greater than the recommended threshold of 0.7, which is the acceptable standard of questionnaire reliability in social science research. Secondly, confirmatory factor analysis (CFA) was performed on the datasets using the AMOS24 statistical software package, to test the discriminant validity of the measures. The results showed that all the indicators were well-fitted, and the hypothesized three-factor model fit was ideal with good discriminant validity. Table 1 below indicates all the model fit statistical measurements for this study.

**Table 1:** Model fit statistics for measurement models.

Measurement	χ^2^/df	RMSEA	CFI	IFI	GFI	TLI
Three-factor-model	1.819	0.056	0.926	0.927	0.892	0.916
Two-factor-model	2.532	0.076	0.860	0.862	0.840	0.843
One-factor-model	3.470	0.097	0.773	0.775	0.781	0.746

*N* = 263. The three-factor model jointly evaluates inclusive leadership, relational silence, and employee innovative behavior on the same factor. The two-factor model combines two factors with inclusive leadership and relational silence and employee innovative behavior on the same factor. The one-factor model employs one of the factors of inclusive leadership, relational silence, and employee innovative behavior on the same factor.

## Results

### Test of common method biases

To avoid the problem of common method bias, anonymous measurements were employed in the questionnaire survey. Afterward, Harman single factor method was used to analyze all the items contained in the inclusive leadership, relational silence, and employee innovation behavior metrics of the questionnaire. Furthermore, the first factor only explained about 35.36 percent of the variance, which is less than the recommended 40 percent threshold indicating that common method bias is not a serious problem in this study (Podsakoff et al., 2003).

### Descriptive statistics

The descriptive statistics of the study comprising an analysis of the mean, standard deviation, and correlation coefficient of the variables that were employed in this research is shown in Table 2. The results revealed that inclusive leadership was negatively correlated with relational silence (*β* = −0.469, *р* < 0.01) and positively correlated with employee innovation behavior (*β* = 0.590, *р* < 0.01), while there exists a significant negative correlation between relational silence and employees’ innovative behavior (*β* = −0.408, *р* < 0.01).

**Table 2:** Descriptive statistics and inter-correlations.

Variables	Mean	SD	1	2	3
1. Inclusive leadership	3.554	0.648	1		
2. Relational silence	2.129	0.524	−0.469^**^	1	
3. Innovative behavior	3.648	0.665	0.590^**^	−0.408^**^	1

*N* = 263.

** *р* < 0.01 (two-tailed).

### Main effect testing

Model 1 and Model 2 are the base models. After adding the independent variables, Model 2 and Model 4 were formed. Model 4 in Table 3 shows that inclusive leadership has a significant positive effect on employee innovation behavior (*β* = 0.589, *р* < 0.001) after controlling for related variables. Hence, the results from the analysis indicate that *Hypothesis 1* was supported, as shown in Table 3 below.

**Table 3:** The results of regression analysis.

	Relational silence	Employees’ innovative behavior			
	Model 1	Model 2	Model 3	Model 4	Model 5
1. Control variables					
Gender	−0.153^*^	−0.129^*^	0.168^*^	0.187^*^	0.160
Age	0.073	0.166^*^	0.012	−0.051	−0.036
Education	−0.080	−0.061	0.058	0.035	0.025
Working time	0.050	0.088	0.047	0.003	0.012
Position level	0.015	−0.024	−0.140^*^	−0.083	−0.086
Job position	−0.013	0.009	−0.018	−0.019	0.054
2. Independent variable					
Inclusive leadership		−0.497^***^		0.589^***^	0.507^***^
3. Mediative variable					
Relational silence					−0.204^**^
R^2^	0.026	0.254	0.069	0.373	0.392
ΔR^2^	0.003	0.233	0.047	0.356	0.373
F	1.147	12.384^***^	3.153^*^	21.646^***^	20.472^***^

*N* = 263. The results presented are the standard beta coefficients of the equation.

* *р* < 0.05.

** *р* < 0.01.

*** *р* < 0.001 (two-tailed).

### Mediating effect testing

Baron and Kenny’s (1986) sequential test was used to verify the mediating effect of the model equation, and the results are shown in Table 3. From Model 2 in Table 3, inclusive leadership had a significant negative effect on relational silence (*β* = −0.497, *р* < 0.001) after controlling for related variables. Therefore, the potency of *Hypothesis 2* could be verified in this study. Going further, based on Model 4, Model 5 was obtained by adding the intermediary variable, which is relational silence. From Model 5, the effect of relational silence on employees’ innovation behavior was significantly negative (*β* = −0.204, *р* < 0.01). expectedly, *Hypothesis 3* was also supported. Interestingly, the influence coefficient of inclusive leadership on employees’ innovative behavior decreased from (*β* = 0.589, *р* < 0.001) to (*β* = 0.507, *р* < 0.001), when relational silence partially mediates the relationship between inclusive leadership and employee innovative behavior. Therefore, *Hypothesis 4* was also fully supported in this study.

## Discussion

From the perspective of relational silence, this paper investigated the impact of inclusive leadership on employees’ innovative behavior. The findings revealed that relational silence has a partial mediating effect in the relationship between inclusive leadership and employees’ innovative behavior; inclusive leadership had a significant positive predictive effect on employees’ innovative behavior; inclusive leadership had a significant negative relationship with relational silence; relational silence had a significant negative relationship with employees’ innovative behavior; The paper explicated the partial mediating role of relational silence in the relationship between inclusive leadership and employee innovation.

Many studies have been conducted to investigate the mechanisms by which inclusive leadership influences employees’ innovative behavior, with Cetinkaya et al. investigating the relationship between inclusive leadership and innovative work behavior, as well as the roles of psychological empowerment and leader-member exchange in this relationship. The findings revealed that inclusive leadership predicted an increase in workers’ innovative work behaviors, with psychological empowerment as a mediation in this relationship (Cetinkaya and Yesilada, 2022). Other mediating variables, such as psychological security (Brown and Treviño, 2006; Kark and Carmeli, 2009; Javed et al., 2019; Wang et al., 2021), creative self-efficacy (Tierney and Farmer, 2002; Javed et al., 2021; Wang et al., 2021), knowledge sharing (Lee et al., 2010), psychological capital (Fang et al., 2019), perceived organizational support (Qi et al., 2019), Error Management Climate and Self-Efficacy (Yuan et al., 2022), and Employee Voice Behavior (Zheng et al., 2022), have also been used in studies to investigate the impact of inclusive leadership on employees’ innovative behavior. The results show that inclusive leadership positively affects employees’ innovative behavior (Javed et al., 2021; Siyal et al., 2021; Wang et al., 2021). Based on the Chinese context, this paper investigates the effect of inclusive leadership on employee innovation behavior. The relationship between the two is consistent with previous research findings and confirms that inclusive leadership and employee innovation behavior are positively related and positively predict employee innovation behavior. However, these studies differ from the mediating variables chosen in this paper. This paper uses relational silence as a mediating variable to clarify the partial mediating effect of relational silence between inclusive leadership and employee innovation behavior for the first time.

The behavior of inclusive leaders is associated with meeting followers’ basic needs for relevance and competence. Meeting these basic needs is associated with increasing employee voice behavior, which, conversely, remains silent. If employees are motivated, they are likely to express their ideas, opinions, and suggestions (Jolly and Lee, 2021). Through mediating effects, professional inspiration negatively impacts employees’ silent behavior, but inclusive leadership moderates the relationship (Xu et al., 2022). The relationship between abusive supervision and employee creative performance is mediated by relational conflict and employee silence (Lee et al., 2022). More research needs to be reported on the relationship between inclusive leadership and relational silence. In our study, there is a significant negative correlation between inclusive leadership and relational silence, and inclusive leadership styles effectively reduce relational silence between leaders and subordinates. Inclusive leader behavior leads employees to believe that the leader is receptive to their differing performance, that the leader can listen to all types of opinions, including opposing voices, and that employees are allowed to express their opinions and expose wrongdoing, reducing the incidence of relational silence between the leader and employees (Brown et al., 2005; Brown and Treviño, 2006; Nembhard and Edmondson, 2006). The accessibility of the leader’s behavior enables employees to actively reflect on their issues and views to the leader actively, thus increasing the opportunities for communication between the leader and employees and thus reducing the occurrence of relational silence (Jolly and Lee, 2021).

Employee silence is negatively related to innovative work behavior (IWB; Maqbool et al., 2019). The highest level of IWB occurs when individuals are absorbed and enjoy their work, with low employee silence, allowing them to exchange ideas and receive the necessary support and resources to facilitate employee innovation. Abusive supervision has a negative impact on creative performance. Employee silence acts as a buffer between abusive supervision and creative performance (Lee et al., 2022). Employee silence has been refined into six dimensions, including relational silence. Relational silence is the behavior of employees who choose to remain silent to protect the organization’s harmonious interpersonal relationship (Brinsfield, 2013). Although research has revealed that employee silence has a detrimental influence on employee innovativeness, no literature has been documented that investigates how inclusive leadership affects employees’ innovative behavior, with relationship silence serving as a mediating variable. Our study reveals that relational silence has a significant negative relationship with employees’ innovative behavior.

We elucidate for the first time that relational silence mediates the effect between inclusive leadership and employee innovation behavior. Employee silence is pervasive in all types of organizations in the Chinese cultural environment. Surveys reveal that over 85% of Employee in the industry admit to being silent on at least some work concerns. Silent behavior hinders innovation, reform, and organizational advancement and development. Inclusive leadership, on the contrary, can reduce the occurrence of relational silence by improving the quality of leadership-employee relationships and creating an environment of good communication and feedback, encouraging employees to express their opinions and ideas, revealing weaknesses and problems in their work, and ultimately inspiring them to solve problems and be innovative. This paper theoretically broadens and enriches the mechanisms of inclusive leadership’s role in influencing employees’ innovative behavior, provides practical guidance for constructing an inclusive leadership style marked by openness, inclusiveness, and accessibility, and lays the theoretical foundation for promoting the reduction of relational silence occurrence and, ultimately, maximizing the evoking of employees’ innovative behavior.

## Conclusion and implications

Consistent with prior studies, as well as the vast literature and empirical evidence that was provided in the previous sections of this study, the main findings of the research reveal that inclusive leadership has a net positive predictive effect on employees’ innovative behavior. Essentially, the more inclusive the leadership style, the more likely it is to stimulate new ideas that make employees willing to try and also enable them to exhibit more innovative behaviors at the workplace. In other words, when employees perceive that their boss has an inclusive style, they are more likely to implement innovative ideas and demonstrate innovative behavior. Therefore, in a fiercely competitive marketplace, enterprises should constantly innovate to maintain and attract new customers, clients, suppliers, distributors, lenders *etcetera*. In the long run, sustainable efforts to foster innovation would also accelerate the pace of firm-wide growth, as well as national growth and development. To make employees’ innovative behavior continue to occur, and to build a relaxed and harmonious organizational atmosphere, developing an inclusive leadership style is therefore important, necessary, and inviolable in a 21st-century workplace.

Unsurprisingly, in this study, the influence of inclusive leadership on relational silence is negative and significant. Consequently, employees might remain silent at work for fear of damaging their relationships with their leaders and colleagues. However, they can change this behavior when their leaders are inclusive — in doing so, makes them more open, effective, and accessible, thereby reducing the frequency and degree of relational silence at the workplace. That said, the empirical analysis conducted to test the veracity of the propositions made by the researcher decisively submits that relational silence has a negative and significant effect on employees’ innovative behavior. Hence, long-term silence can cause employees to pay less attention to problems in the organization, as well as make them give less feedback on negative information. This can make an organization miss out on an opportunity to solve problems within a firm, as well as lose an opportunity to innovate and change.

In addition, this research uncovered that the influence mechanism of inclusive leadership on employees’ innovative behavior does not occur in isolation, which is because relational silence plays a mediating role in this relationship. More so, this implies that the inclusive style employed by inclusive leaders puts employees in a more relaxed state, as well as helps to reduce the occurrence of relational silence. Hence, employees dare to express their opinions and ideas, and also actively send feedback for queries relating to the problems in the production and management of the organization, thus promoting the emergence of employees’ innovative behavior. In other words, when employees perceive their leaders to be inclusive, their initial fear of lowering their leaders by expressing their thoughts diminishes over time. Likewise, as the quality of the relationship between inclusive leaders and their employees improves, their fear towards them would diminish as a result of the discordance that occurs between employees, while the psychological security amongst them would be strengthened. Thus, the staff working under inclusive leaders will be more actively involved in their work, and constantly seek breakthrough and innovative work ideas, *ceteris paribus*.

In conclusion, for organizations, it is necessary to create a relaxed and harmonious organizational environment, which entails building an inclusive, active, open-minded organizational culture, so that employees and managers can communicate actively and effectively. Thus, when organizations are willing to listen to the views and suggestions of their employees, it reduces the impact of the external environment on employees’ silent behavior. For managers, it is absolutely important to develop an inclusive leadership style, care for and respect employees, treat employees with an open and accessible attitude, praise, promote, establish and improve communication channels, and actively listen to employees’ innovative ideas, to meet the individual needs of employees. Correspondingly, when employees encounter work problems, managers should endeavor to try to solve their problems in the innovation process. Similarly, when employees have innovative ideas and innovative results, they should be given both material and non-monetary incentives — to spur them to do more. For employees, they should be made to understand from the get-go that employee silence is detrimental to the development of their work, while cooperation with others (i.e., teamwork) and the development of the enterprise is essential for organizational success. Hence, employees should be brave enough to freely explore new things, as well as actively express new ideas and thought processes, when using available enterprise resources to intervene promptly in the performance of organizational tasks, as well as during the implementation of new work ideas at the workplace.

### Limitations and future research direction

First of all, since the source of the sample population is mainly from in-service leaders, and employees of enterprises that work in the Guangdong province of China, future studies can be expanded in scope to include regions, countries, and continents. This would enhance the generalizability, validity, and reliability of such a study. Secondly, because the number of samples collected was not adequate or large enough to warrant the applicability of the findings of this study to a similar context, the number of samples in future studies can be significantly increased to improve the explanatory power of the ensuing predictions thereafter. Finally, given the opacity of the phenomenon and the sparseness of research in this area, there is a fundamental lack of literature research materials on relational silence. This problem, therefore, limits the researcher’s ability to gather either enough references or reference materials, as well as limits the ability of the researcher to perform the critical task of providing the theoretical basis for the study. Thus, we recommend a longitudinal study on relational silence for future studies in this area.

## Data availability statement

The original contributions presented in the study are included in the article/supplementary material, further inquiries can be directed to the corresponding author.

## Author contributions

G-fW responsible for conceptualisation, methodology, software, and writing-original manuscript preparation. ML participated in writing-review, editing, and funding. All authors contributed to the article and approved the submitted version.

## Funding

This work was funded by the Education Department of Guangdong Province, China (2020ZDZX3027), the Zhongshan Municipal Science and Technology Bureau, China (2019B2013), and the University of Electronic Science and Technology of China Zhongshan Institute, China (KJYS202206).

## Conflict of interest

The authors declare that the research was conducted in the absence of any commercial or financial relationships that could be construed as a potential conflict of interest.

## Publisher’s note

All claims expressed in this article are solely those of the authors and do not necessarily represent those of their affiliated organizations, or those of the publisher, the editors and the reviewers. Any product that may be evaluated in this article, or claim that may be made by its manufacturer, is not guaranteed or endorsed by the publisher.
